# Effect of *Celastrus orbiculatus* in inhibiting *Helicobacter pylori* induced inflammatory response by regulating epithelial mesenchymal transition and targeting miR-21/PDCD4 signaling pathway in gastric epithelial cells

**DOI:** 10.1186/s12906-019-2504-x

**Published:** 2019-04-29

**Authors:** Yaodong Zhu, Lei Liu, Lei Hu, Wenqing Dong, Mei Zhang, Yanqing Liu, Ping Li

**Affiliations:** 1 Chinese Integrative Medicine Oncology Department, First Affiliated Hospital of Medical University of Anhui, Hefei, 230000 Anhui China; 2 General Surgery Department, First Affiliated Hospital of Medical University of Anhui, Hefei, 230000 Anhui China; 3 Emergency Department, First Affiliated Hospital of Medical University of Anhui, Hefei, 230000 Anhui China; 4 Institute of Combining Chinese Traditional and Western Medicine, Medical College, Yangzhou University, Yangzhou, 225009 Jiangsu China

**Keywords:** *Celastrus orbiculatus*, Inflammatory response, Epithelial mesenchymal transition, PDCD4, miR-21

## Abstract

**Background:**

The extract of *Celastrus orbiculatus* (COE) have been studied for anti-*Helicobacter pylori* (*H. pylori*) activity and anti-cancer effects in vitro and in vivo. However, the molecular mechanism by which COE inhibits *H. pylori*-induced inflammatory response has not been fully elucidated so far.

**Methods:**

The effects of COE on viability, morphological changes, inflammatory cytokine secretion, protein and mRNA expression were analyzed by MTT assay, enzyme-linked immunosorbent assay (ELISA), immunofluorescence, western blot and real-time PCR (RT-PCR), respectively. The methylation level of programmed cell death 4 (PDCD4) promoter was investigated by methylation-specific PCR.

(MSP) .

**Results:**

COE effectively inhibited the *H.pylori*-induced inflammatory response by regulating epithelial-mesenchymal transition (EMT). The methylation level of PDCD4 promoter was suppressed by COE, which increased the expression ofPDCD4. Moreover, COE could inhibit microRNA-21 (miR-21) expression, as shown by an enhancement of its target gene PDCD4. Furthermore, both miR-21 over-expression and PDCD4 silencing attenuated the anti-inflammatory effect.

of COE.

**Conclusions:**

COE inhibits *H. pylori* induced inflammatory response through regulating EMT, correlating with inhibition of miR-21/PDCD4 signal pathways in gastric epithelial cells.

**Electronic supplementary material:**

The online version of this article (10.1186/s12906-019-2504-x) contains supplementary material, which is available to authorized users.

## Background

*Helicobacter pylori* (*H.pylori*) is related to gastrointestinal illnesses such as chronic gastritis, gastric ulcer and gastric carcinomas [1]. It is believed to be associated with infection-initiated inflammatory response, which is characterized by altered cytokine production in gastric epithelial cells [2].

Epithelial-mesenchymal transition (EMT) is thought to be involved in the inflammatory response in many inflammatory diseases, which includes down-regulation of epithelial cell markers and up-regulation of mesenchymal markers [3]. It has been reported that *H. pylori* infection not only damages the gastric mucosa, but also causes the inflammatory response by inducing EMT [4]. Thus, targeting EMT could become a promising therapeutic approach for inhibiting inflammatory responses.

Programmed cell death 4 (PDCD4), a pro-inflammatory tumor suppressor protein, may play a vital role in the inflammatory diseases [5]. It is considered that decreased PDCD4 expression would promote inflammatory response that would promote the development of gastritis [6]. Recent studies suggest that microRNA-21 (miR-21) functions as a pro-inflammatory regulator and oncomiR between inflammation and cancer [7]. Moreover, miR-21 may be involved in inflammatory response by down-regulating the expression level of PDCD4 [8]. Therefore, miR-21/PDCD4 signaling pathway were focused on in our study.

*Celastrus orbiculatus* has been widely used in Chinese folk medicine for treatment of many diseases, including arthritis and other inflammatory diseases [9]. It was found that the ethyl acetate extract of *Celastrus orbiculatus* (COE) displays anti-*H. pylori* activity and anti-cancer effects in vitro and in vivo [10]. Although COE has been reported in various researches, the mechanism by which COE on *H. pylori* induced inflammatory responses in gastric epithelial cells has not been fully elucidated.

Therefore, this study reports that COE can inhibit *H. pylori* induces inflammatory response by regulating EMT and miR-21/PDCD4 signaling pathway. The results indicated that COE may have potential therapeutic potential as an anti-inflammatory drug in *H. pylori*-infected gastric epithelium.

## Methods

### Chemicals and reagents

*H. pylori* Sydney strain 2000 (SS2000) was obtained from the National Centers for Disease Control. DMEM and fetal bovine serum (FBS) was acquired from Gibco-BRL (Gaithersburg, MD, USA). Human interleukin 6 (IL-6), IL-8, and tumor necrosis factor alpha (TNF-α) mini enzyme-linked immunosorbent assay (ELISA) development kit were obtained from MAbtech AB (Nacka Strand, Sweden). Antibodies against E-cadherin, N-cadherin, Vimentin and GAPDH were purchased from Cell Signaling Technology (Beverly, MA, USA). Antibodies against PDCD4 was purchased from Abcam (Cambridge, MA, USA). Other chemicals used were of analytical grade from commercial sources.

### Preparation of COE

The herbal pieces of *Celastrus orbiculatus* were purchased from Guangzhou Zhixin Pharmaceutical Co Ltd. (Guangzhou, China). The preparation procedure of COE has been described previously [11]. The chemical constituents from the stems of *Celastrus orbiculatus* were investigated by High Performance Liquid Chromatography (HPLC) assay (Additional file 1). The resultant COE micropowder was dissolved in dimethyl sulfoxide (DMSO, Sigma-Aldrich Co., St Louis, MO, USA).

### Cell culture

The human gastric epithelial cell line GES-1 was obtained from Shanghai Institute of Biochemistry and Cell Biology, Chinese Academy of Sciences (Shanghai, China). Cells were cultured in DMEM Medium supplemented with 10% FBS at 37 °C with 5% CO_2_. *H. pylori*, suspended in DMEM, was added to cells at a bacterium/cells ratio of 200:1. The cells were treated by COE for 24 h after *H. pylori* stimulation for 2 h.

### MTT assay

GES-1 cells were planted in a 96-well plate and cultured overnight. The cells were treated with different concentrations of COE (10, 20, 40 and 80 μg/mL) for 24, 48 and 72 h. After incubation with MTT solution for 4 h, the absorbance was measured at 490 nm in a microplate reader.

### Enzyme-linked immunosorbent assay (ELISA)

The assessment of the IL-6, IL-8 and TNF-α levels in the the supernatant fluid of cells was performed by ELISA, using ELISA kits according to the manufacturer’s instructions. The absorbance at 450 nm was measured.

### Immunofluorescence

Cells were fixed with paraformaldehyde and then blocked with Triton X-100 and serum for 30 min. Then, the cells were incubated with primary antibodies at 4 °C overnight. After washed with PBS for 3 times, the secondary antibodies were incubated at 37 °C for 1 h. The nuclei were dyed with 4,6-diamidino-2-.

phenylindole (DAPI) (Bioworld Technology, St Louis, USA). Image were obtained under fluorescence microscope at × 400 magnification.

### Western blot analysis

Proteins were separated by SDS–PAGE gel and transferred into nitrocellulose membranes (Millipore, Bedford, MA). The membrane was blocked with 5% non-fat milk for 2 h. The membranes were washed and secondary antibody was added to the system. Immunoreactive protein bands were detected using an Molecular Imager Chemi Doc XRS System (Bio-Rad). The bands from western blotting were quantified by Quantity One analysis software (Bio-Rad).

### Real-time PCR (RT-PCR)

Total RNAs were isolated according to the manufacturer’s instructions. Primers were obtained from Shanghai Sangon Biological Engineering Technology and Services (Shanghai, China) and their sequences were: E-cadherin, forward primer 5′-ATG AGG TCG GTG CCC GTA TT-3′, reverse primer 5′- CGT TGGT CTT GGG GTC TGT GA-3′; N-cadherin, forward primer 5′-TCA GTG GCG GAG ATC CTA C-3′, reverse primer 5′-GTG CTG AAT TCC CTT GGC TA-3′; Vimentin, forward primer 5′-AAG CAG GAG TCA AAC GAG TA-3′, reverse primer 5′-GTT GGC AGA GGC AGA GAA AT-3′; PDCD4, forward primer 5′-AAA GGC GAC TAA GGA AAA CTC ATC-3′, reverse primer 5′-GCC TAT CCA GCA ACC TTC CCT-3′; GAPDH, forward primer 5′-CTC AAC TAC ATG GTC TAC ATG TTC CA-3′, reverse primer 5′-CTT CCC ATT CTC AGC CTT GAC T-3′. RT-PCR was conducted using an ABI 7500 Real-Time PCR System (Applied Biosystems). GAPDH was used as an internal normalization control. The results were calculated using the 2^-∆∆Ct^ method.

### Methylation-specific PCR (MSP) assay

Total DNA was extracted using the Universal Genomic DNA Extraction Kit Ver.3.0 (TaKaRa). Non-methylated and methylated regions of DNA were amplified after treatment with sodium bisulfite. The PDCD4 gene methylated primer sequences used were as follows: 5′-TCG TCG TTA CGA TTG GTT AGT C -3′ (forward) and 5′-GAA AAA TCT CTA ACC CTT CTC GC -3′ (reverse). The PDCD4 gene non-methylated primer sequences used were as follows: 5′- GGT TTT GTT GTT ATG ATT GGT TAG TT -3′ (forward) and 5′-CAA AAA ATC TCT AAC CCT TCT CAC T -3′ (reverse). DNA methylation analysis was conducted following methylation specific PCR amplification. The products of PCR were detected by 1.5% agarose gel electrophoresis.

### Cell transfection

miR-21 precursor and negative control precursor miRNA were purchased from GenePharma (Shanghai, China). PDCD4 small interfering RNA (siRNA) and scrambled control siRNA were obtained from Santa Cruz Biotechnology. They were similarly transfected using Lipofectamine 2000 (Invitrogen, San Diego, CA, USA).

### Statistical analysis

Date were shown as the means ± SD. Unpaired two-tailed Student’s *t*-test was used to compare two independent groups. One-way analysis of variance (ANOVA) was used to compare more than two groups. SPSS for Windows, version 17 (SPSS, Inc.) was used for all statistical analyses, and *P* < 0.05 was considered to indicate a statistically significant difference.

## Results

### Effect of COE on *H. pylori* infection-induced cell morphological changes

**Fig. 1:**
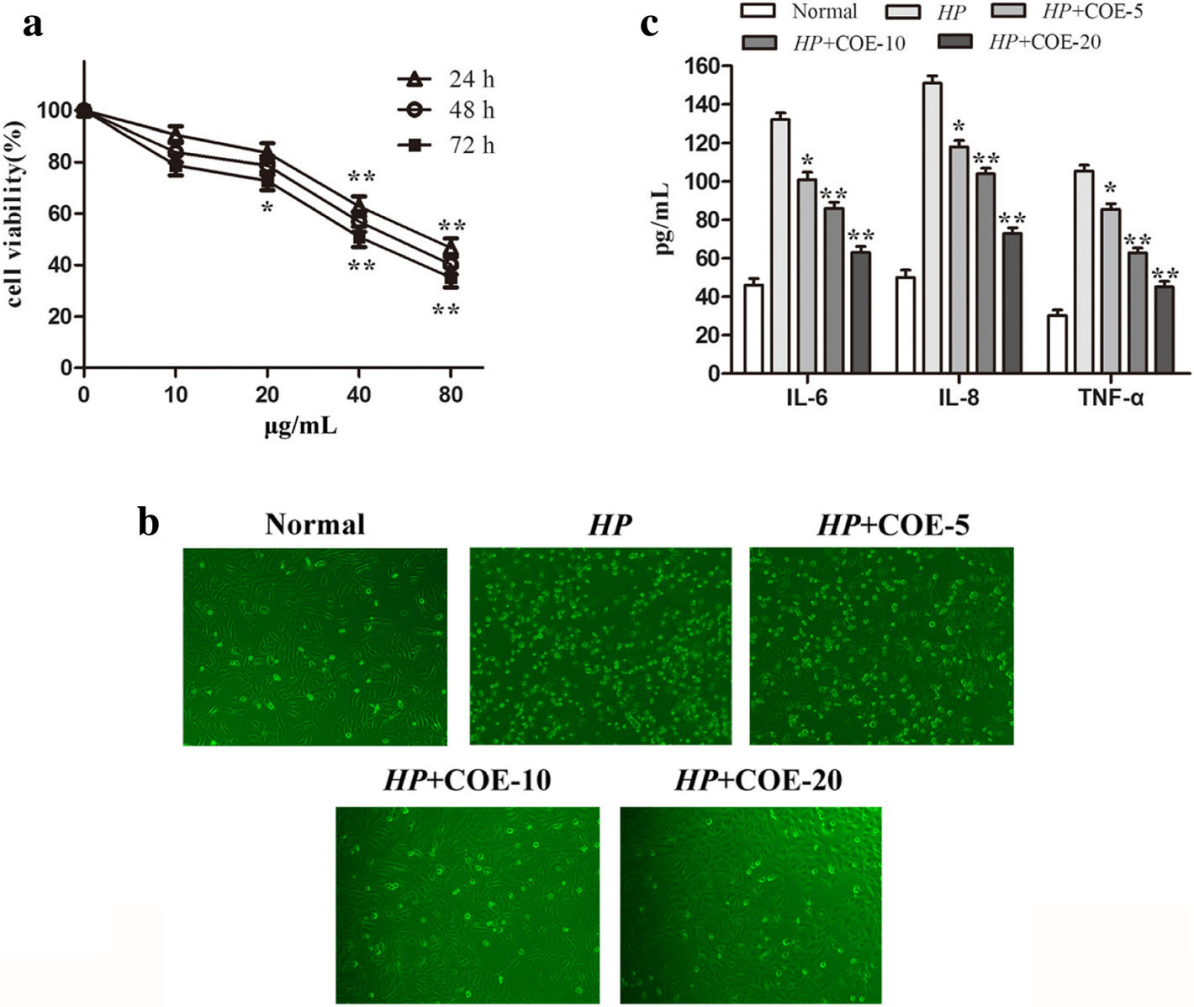
Effect of COE on *H. pylori* infection-induced cell morphological and inflammatory cytokine changes**.** (**a**) The effects of COE toxicity on cell viability of GES-1 cells after 24, 48, 72 h culture. The cell viability showed the results of three separate experiments as mean ± SD, *P < 0.05, **P < 0.01, vs 0 μg/mL. (**b**) The effects of COE on *H. pylori*-infected cell morphological changes. Representative pictures of cells were obtained under a microscope at × 200 magnification. (**c**) The effects of COE on *H. pylori*-induced pro-inflammatory cytokine changes. ELISA was applied to test the levels of IL-6, IL-8 and TNF-α. Data are presented as mean ± SD of three separate experiments, *P < 0.05, **P < 0.01 compared with the *H. pylori*-infected group

COE inhibited the viability of GES-1 cells in a concentration and time dependent manner. No obvious change in cell viability was observed at 10 and 20 μg/mL of COE (Fig 1a). Therefore, the non-cytotoxic concentrations (below 20 μg/mL) of COE was chosen in further experiments. The appearances of the GES-1 cells are polygonal or fusiform shape. After 24 h co-culture with *H.pylori*, it transformed from multiangular to round or irregular shapes of various sizes, with disrupted cell walls and cytoplasmic leakage. Interestingly, the morphology changes were noticeably suppressed by pretreatment with COE (Fig.1b).

### Effect of COE on *H. pylori* infection-induced inflammatory cytokine changes

The levels of pro-inflammatory cytokines IL-6, IL-8 and TNF-α may serve as sensitive and responsive biomarkers of change in *H. pylori*-induced inflammatory response [12]. As shown in Fig.1c, levels of IL-6, IL-8 and TNF-α were obviously higher in *H. pylori* infection group than in normal group. After treatment with different concentrations of COE, the levels were less when compared with *H. pylori* infection group. It significantly decreased in the 20 μg/mL COE group.

### Effect of COE on EMT-related gene expression

**Fig. 2:**
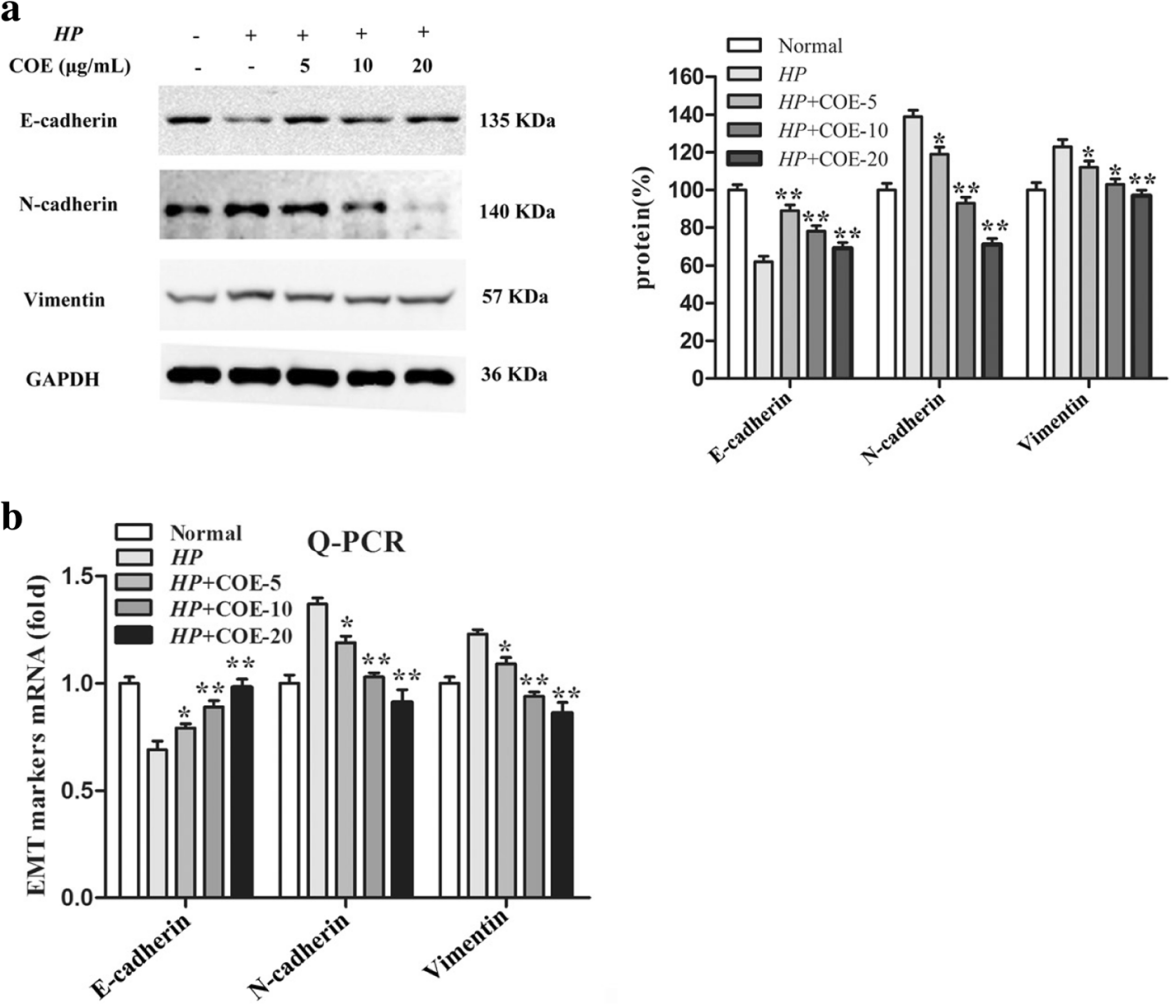
Effect of COE on *H. pylori* infection-induced EMT-related gene expression. (**a**) Protein expression levels of E-cadherin, N-cadherin and vimentin were evaluated by western blot. GAPDH was served as an internal control of protein level. The relative density of EMT markers was normalized to GAPDH, which was determined by densitometric analysis. (**b**) Relative mRNA levels of EMT-related gene were examined by RT-PCR. The data was analyzed using the 2^-∆∆Ct^ method. Each experiment from three independent experiments and values are expressed as means ± SD of three independent experiments. *P < 0.05, **P < 0.01, compared to *H. pylori* infection-induced group

**Fig. 3:**
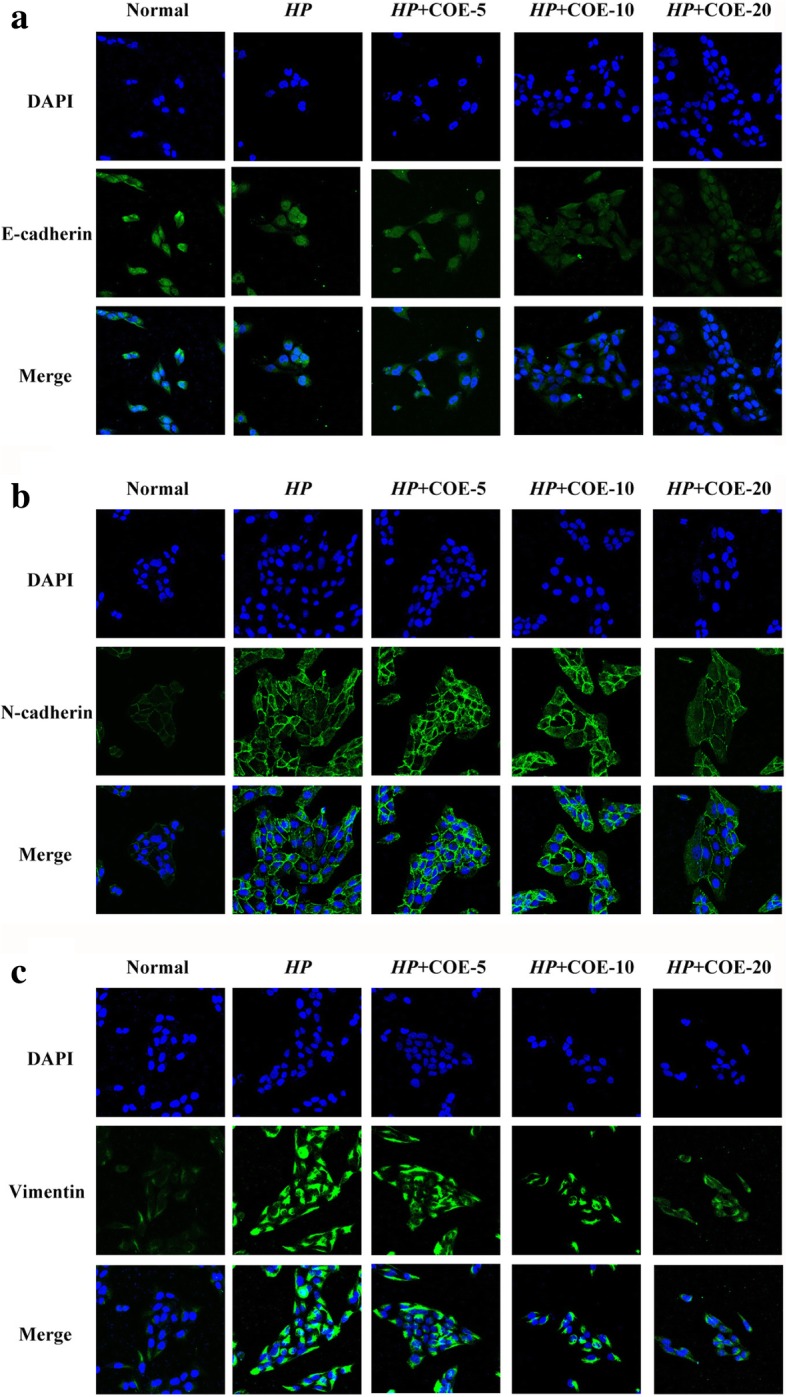
Immunofluorescence microscopy analysis of the localization and expression of EMT-related gene in *H. pylori* infection-induced GES-1 cells treated with or without COE. Representative pictures of cells were obtained under a microscope at × 400 magnification

Our date showed that *H. pylori* infection-induced loss of epithelial marker E-cadherin, while up-regulation of mesenchymal markers N-cadherin and Vimentin at both protein and mRNA levels. However, pretreatment with COE effectively prevented these variations in a concentration-dependent manner (Fig. 2a-b). In addition, immunofluorescence staining also revealed that EMT-related gene expression were in keeping with the above research work (Fig. 3a-c). Taken together,

these findings showed that COE inhibited the effects of *H. pylori* infection-induced EMT in gastric epithelial cells.

### Effect of COE on methylation status and expression of PDCD4 gene

**Fig. 4:**
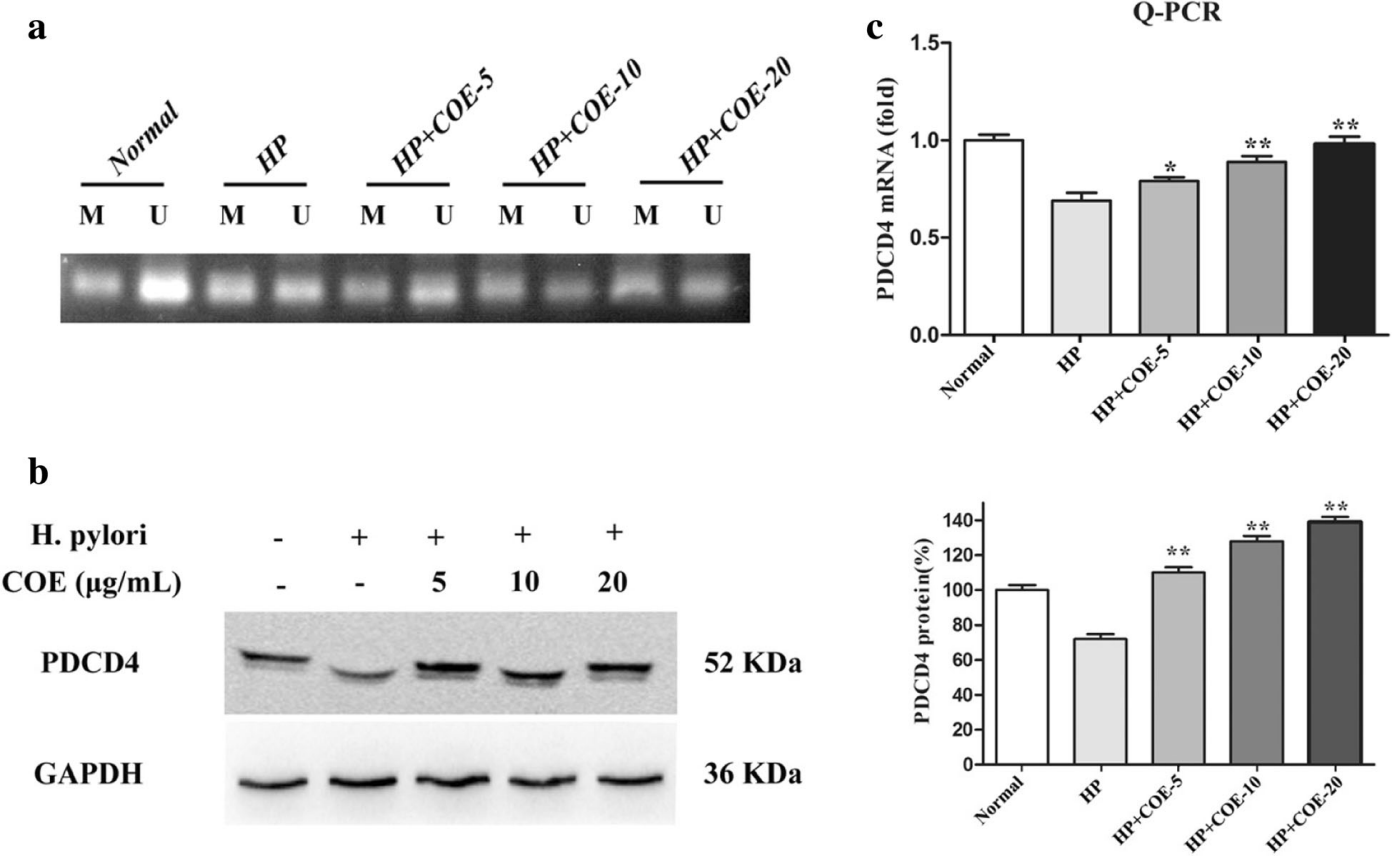
Effect of COE on *H. pylori* infection-induced methylation status and expression of PDCD4 gene. (**a**) The methylation status of the PDCD4 promoter induced by COE determined via the MSP assay. U-non-methylated product; M-methylated product. (**b**) PDCD4 protein levels after COE treatment was detected by western blot analysis. The relative density was normalized to GAPDH, which was determined by densitometric analysis. (**c**) PDCD4 mRNA levels after COE treatment was detected by RT-PCR. The data was analyzed using the 2^-∆∆Ct^ method. Each experiment from three independent experiments and values are expressed as means ± SD of three independent experiments. *P < 0.05, **P < 0.01, compared to *H. pylori* infection-induced group

Recent studies have shown that the expression of PDCD4 is frequently silenced or reduced as a result of hypermethylation in tumor inflammatory microenvironment [13]. Therefore, the methylation status of the PDCD4 promoter region was investigated. The methylation level of PDCD4’s promoter was higher in *H. pylori* infection group than in normal group. However, after the treatment of COE, the promoter of PDCD4 had demethylation (Fig.4a). In addition, the effect of COE on PDCD4 demethylation was subjected, with western blotting and RT-PCR assay, which supported these findings (Fig.4b-c). These results demonstrated that COE significantly up-regulated the expression of PDCD4 by influencing the methylation status of its promoter.

### COE inhibits *H. pylori*-induced miR-21 elevation and PDCD4 reduction

**Fig. 5:**
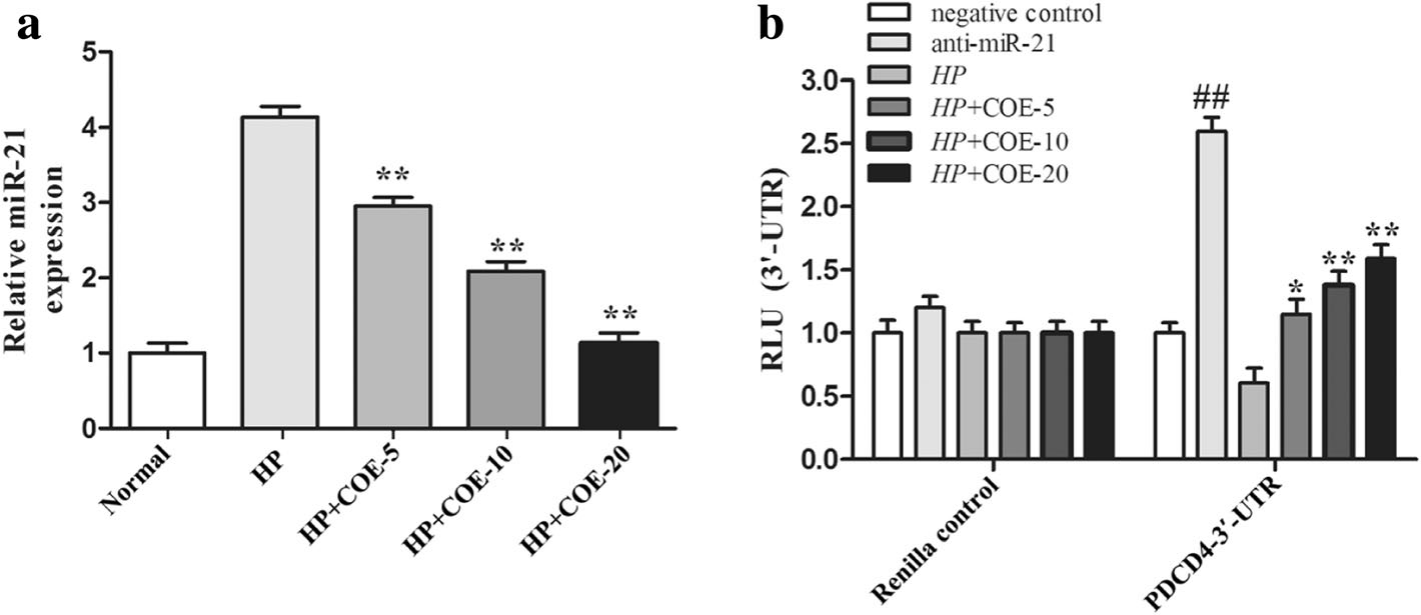
COE inhibits *H. pylori*-induced miR-21 elevation and PDCD4 reduction. (**a**) GES-1 cells were treated by COE for 24 h after stimulating by *H. pylori* for 2 h. The miR-21 expression was quantitated by realtime-PCR. (**b**) COE ameliorates the *H. pylori* -induced inhibition of PDCD4 3′-UTR reporter activity. GES-1 cells were transfected with renilla reporter construct (pGL3-PDCD4_3’-UTR), miR-21 inhibitor (100 nM), negative control (100 nM), and pGL3-promoters and treated with *H. pylori* for 2 h in the presence of COE (20 μg/mL). Cellular lysates were subjected to a luciferase reporter assay as described in Materials and Methods. The results are expressed as relative activity (relative luminescence units (RLU)) normalized to the luciferase activity in the vector control cells without treatment. Data presented in the bar graphs are the mean ± SD of three independent experiments. *P < 0.05, **P < 0.01, compared to *H. pylori* infection-induced group. ##*P* < 0.01, compared to negative control group

Increasing evidence indicated that miR-21 may serve as a key mediator of the anti-inflammatory response [14]. The data from the present study revealed that COE inhibited the elevation of miR-21 after *H. pylori* infection in a dose-dependent manner (Fig. 5a). Moreover, as shown in Fig. 5b, cells were cotransfected reporter construct containing the wild-type miR-21 binding site within the PDCD4 3′ UTR or a mutated miR-21 binding site for luciferase reporter assay. The results indicate that PDCD4 luciferase activity increased in cells incubated with 20 μg/mL COE, suggesting that COE modulates PDCD4 expression via miR-21.

### Over-expression of miR-21 and knockdown of PDCD4 impairs the anti-inflammatory effect of COE

**Fig. 6:**
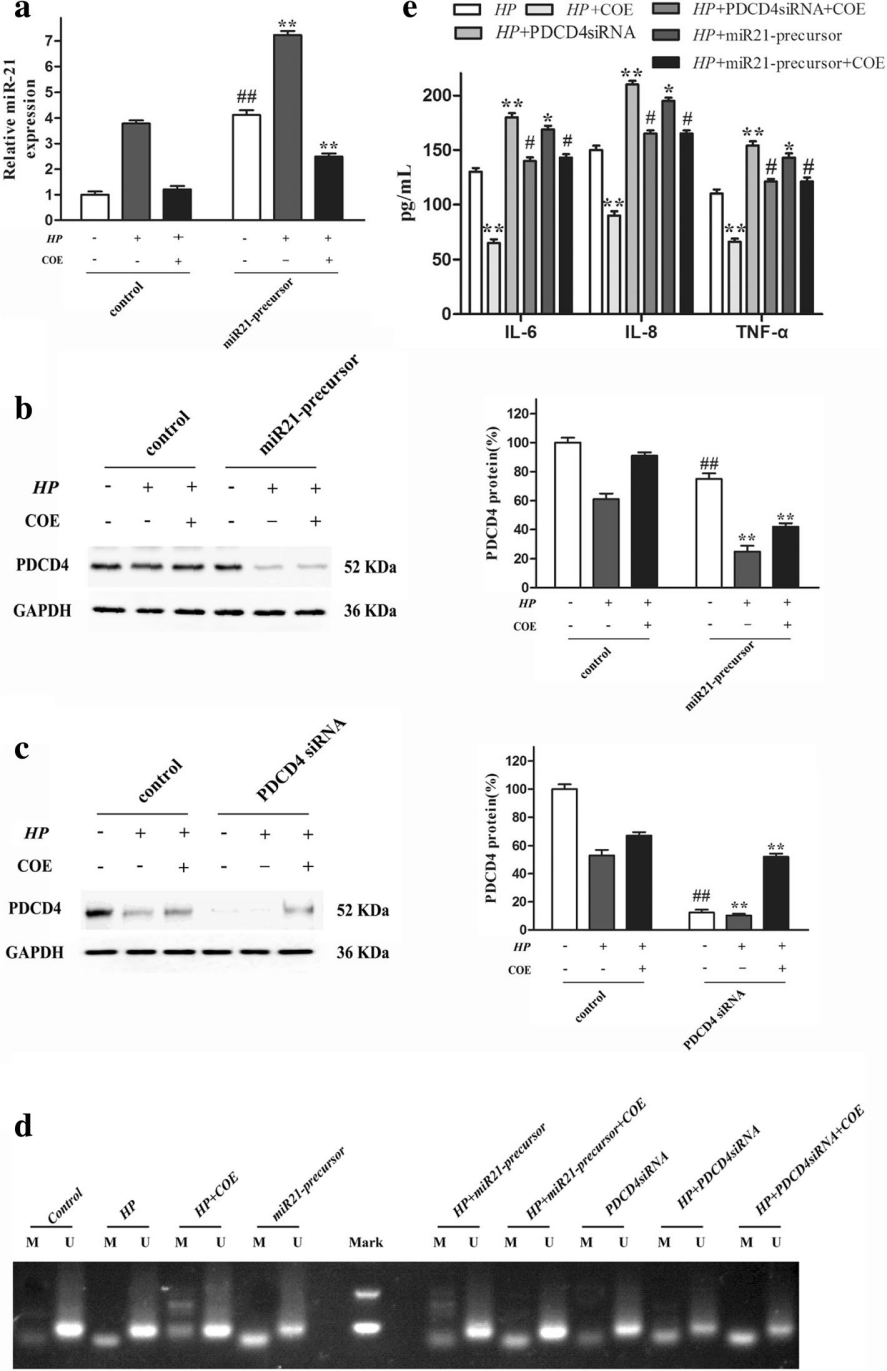
Over-expression of miR-21 and knockdown of PDCD4 impairs the anti-inflammatory effect of COE. GES-1 cells were pre-transfected with miR-21 precursor, PDCD4 siRNA and then exposed to COE for 24 h. (**a**) The relative miR-21 level was determined by RT-PCR. (**b**-**c**) Cell lysates were prepared to determine the protein level of PDCD4 using western blot analysis. The expression of PDCD4 was determined by western blot. (**d**) The MSP assay was performed to evaluate the correlation between the PDCD4 methylation status. (**e**) The anti-inflammatory effect of COE was determined by testing the levels of IL-6, IL-8 and TNF-α. Data are presented as mean ± SD of three separate experiments, *P < 0.05, **P < 0.01, compared to *H. pylori* infection-induced group, #*P* < 0.05, ##*P* < 0.01, compared to transfected with miR-21 precursor or PDCD4 siRNA group.

To evaluate the role of miR-21/PDCD4 signaling pathway in the anti-inflammatory effect of COE, miR-21 precursor were designed and performed. As shown in Fig. 6a, transfection with miR-21 precursor significantly increased miR-21 expression levels, comparing with control cells. Furthermore, over-expression of miR-21 significantly suppressed the COE-induced increase of PDCD4 expression levels, compared with the group treated with COE (Fig. 6b). Moreover, similar findings were observed when PDCD4 was knocked down in GES-1 cells exposure to COE (Fig. 6c). In addition, the methylation status of the PDCD4 promoter was high and the production of pro-inflammatory cytokines was significantly increased in GES-1 cells treated with COE and miR-21 precursor or PDCD4 siRNA, compared with the group treated with COE alone (Fig. 6d-e). These results suggested that miR-21 over-expression and PDCD4 silencing significantly change the methylation status of PDCD4 promoter and attenuated the anti-inflammatory effect in GES-1 cells after treatment with COE.

## Discussion

*H.pylori* colonizes the stomachs of more than half of the world’s population, and the infection continues thought to play a key role in the pathogenesis of a number of gastroduodenal diseases [15]. A triple therapy with proton pump inhibitors and 2 antibiotics is usually effective for treating *H. pylori* infection. However, the increased resistance of *H. pylori* has significantly reduced its eradication rate [16]. Therefore, it is important to develop an alternative methods for eradicating *H. pylori* infections.

In recent years, Chinese herbal medicine has been shown to exhibit bacteriostatic effects on *H. pylori* [17]. Chang et al. found that geniposide, a bioactive extract of Gardenia, could obviously reduce *H. pylori* infections by interfering with the growth and virulence as well as attenuating the inflammatory response [18]. Nagata et al. reported that successful eradication of *H. pylori* with a herbal medicine, goshuyuto (Wu Zhu Yu Tang), prevents relapse of peptic ulcer disease [19]. Therefore, it is expected that herbal products may be promising drugs against *H. pylori* infection and may be able to decrease the risk of gastric cancer.

COE is the product isolated from the stem of *Celastrus orbiculatus*, a Chinese traditional herb [20]. In a pervious study we identified COE as a novel antibacterial agent with anti-*H. pylori* activity in vivo [10]. However, recent reports on experimental data regarding specific agents that reduce *H. pylori* infections and inhibit inflammatory response are limited. In this study, the effect of COE on *H. pylori*-induced inflammatory response in gastric epithelial cells has been discussed.

It is widely accepted that *H. pylori* colonises the gastric mucosa and induces the expression of variable pro-inflammatory cytokines, such as, IL-6, IL-8 and TNF-α, which were all associated with inflammatory response [21]. In this study, we found that COE inhibit the inflammatory process through the inhibition of IL-6, IL-8 and TNF-α release in infected GES-1 cells with *H. pylori*, which strongly suggested that COE exerts anti-inflammatory effects in vitro.

EMT is a common physiological phenomenon, which closely associated with embryonic development, wound healing, tissue regeneration, inflammatory response, as well as cancer progression [22]. In the past several years, EMT had emerged as one of the most interesting topics in the field of inflammatory response, and had caused widespread concern [23]. EMT is always accompanied by the changes of related gene, such as down-regulation of E-cadherin and upregulation of N-cadherin and vimentin [4]. In this study we demonstrated that *H. pylori*-infected induced EMT process in GES-1 cells. COE significantly reduced the expression of N-cadherin and Vimentin, and effectively increased E-cadherin expression. Taken together, these data suggest that COE can inhibit *H. pylori* induced inflammatory response possibly through inhibition the occurrence of EMT.

Recent advances have highlighted the importance of epigenetic alterations in addition to genetic changes in the inflammatory response [24]. To date, DNA methylation is the most commonly studied epigenetic mechanism, which is involved in *H. pylori* infection-induced inflammatory environment [25]. PDCD4 is a new tumor suppressor gene and involved in the EMT process induced by *H. pylori* [26]*.* It has been reported that reduction in PDCD4 protein expression has previously been reported to be associated with abnormal promoter methylation [27]. The present study showed that *H.pylori*-infected promoted PDCD4 methylation, which might lead to reduced expression of PDCD4. COE could upregulated the expression of PDCD4 through inhibition of PDCD4 methylation. Therefore, we speculate that COE might inhibit inflammatory response by reducing PDCD4 gene methylation.

miRNAs modulate gene expression post-transcriptionally by negatively regulating the translation of mRNA to protein [28]. miRNA-21 has a significant role in inflammatory process, cancer progression, and metastasis. Additionally, miR-21 binds to the 3′-UTR of tumor suppressor PDCD4 and suppresses its translation [8]. Therefore, miR-21/PDCD4 signaling pathway was considered as potential targets for novel cancer prevention or anti-inflammatory therapies.

The results of the present study demonstrated that COE treatment reduced miR-21 promoter activity and expression in a dose-dependent manner, and induced the expression of PDCD4, which is a target of miR-21. In support of the hypothesis that regulation of the miR-21/PDCD4 signaling pathway is responsible for the anti-inflammatory activity of COE, we noted that over-expression of miR-21 and knockdown of PDCD4 attenuated inflammatory response in GSE-1 cells after treatment with COE.

## Conclusion

Overall, our data show that COE exerts anti-inflammatory effects on.

*H. pylori* infection induced EMT process in gastric epithelial cells, which is associated with derepression of PDCD4 via downregulation of miR-21. These results suggest that COE may have potential therapeutic benefits as an anti-inflammatory drug for gastritis, ulcers and gastric cancer.

## Additional file

Additional file 1: Analysis of various active compounds in COE was performed using HPLC assay. (DOC 474 kb)

## Abbreviations

COE: extract of *Celastrus orbiculatus*
DAPI: 4, 6-diamidino-2-phenylindole
DMSO: dimethyl sulfoxide
ELISA: enzyme-linked immunosorbent assay
EMT: epithelial mesenchymal transition
MSP: methylation-specific polymerase chain reaction
PDCD4: programmed cell death 4
TNF-α: tumor necrosis factor alpha
