# Supplementary material for: Effect of Celastrus orbiculatus in inhibiting Helicobacter pylori induced inflammatory response by regulating epithelial mesenchymal transition and targeting miR-21/PDCD4 signaling pathway in gastric epithelial cells
Source: BMC Complement Altern Med. 2019 Apr 29;19:91. doi: 10.1186/s12906-019-2504-x (PMC6489279; doi:10.1186/s12906-019-2504-x)
Supplement: Supplementary file 1 — Analysis of various active compounds in COE was performed using HPLC assay. (DOC 474 kb) [file 12906_2019_2504_MOESM1_ESM.doc]

**Additional file 1.**


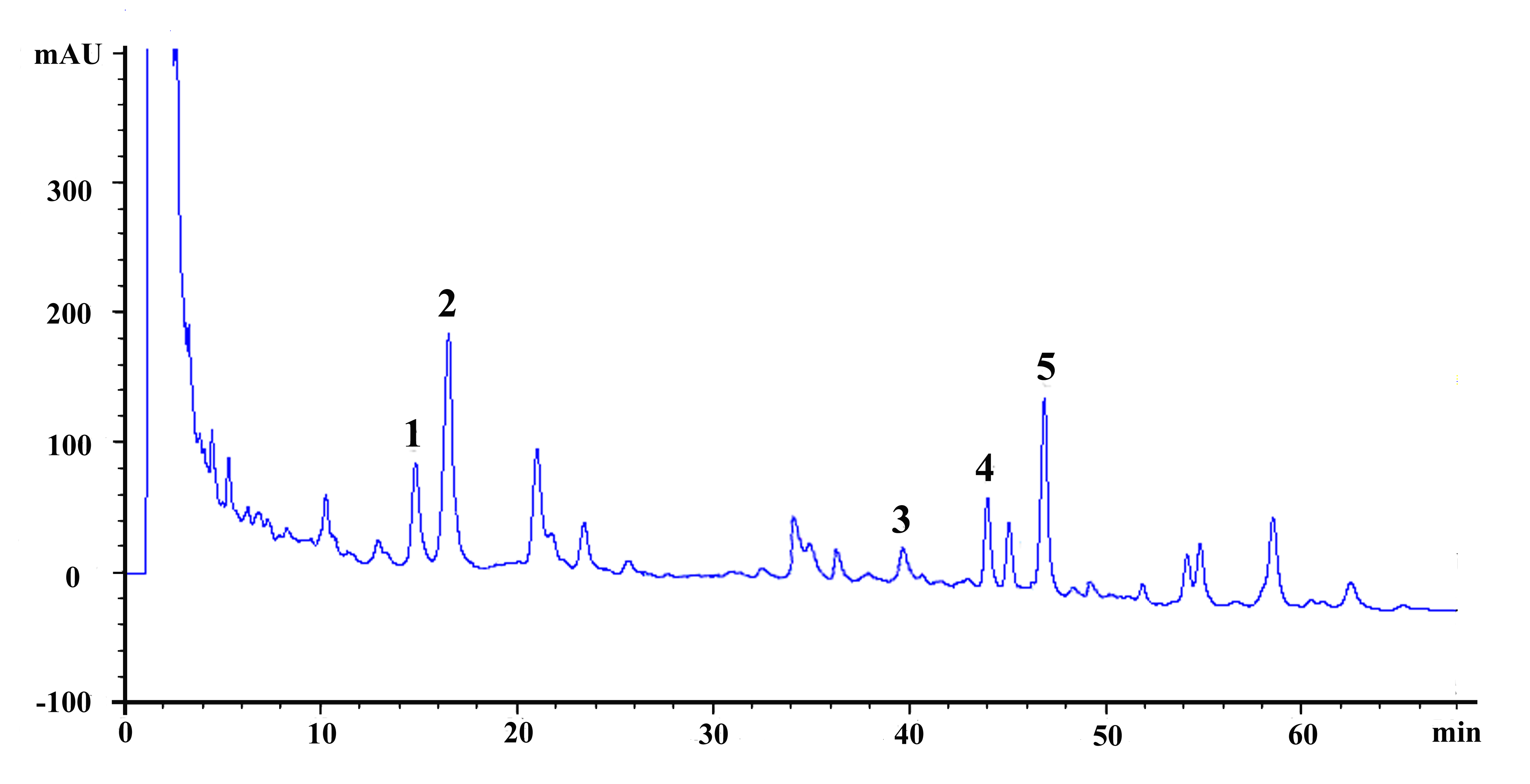


**Additional file 1.** Analysis of various active compounds in COE was performed using HPLC assay. Compounds are identified by number as follows: 23-hydroxybetulonic acid (1), 23-hydroxy-3-oxoolean-12-en-28-oic acid (2), oleanolic acid (3), 3-oxo-24-norolean-12-en-28-oic acid (4) and wiforlide B (5).
